# Validity of an inertial system for measuring velocity, force, and power during hamstring exercises performed on a flywheel resistance training device

**DOI:** 10.7717/peerj.10169

**Published:** 2020-10-09

**Authors:** Rodrigo Martín-San Agustín, Mariana Sánchez-Barbadora, José A. García-Vidal

**Affiliations:** 1 Department of Physiotherapy, University of Valencia, Valencia, España; 2 Department of Physiotherapy, University of Murcia, Murcia, Spain

**Keywords:** Inertial training, Exercise testing, Inertial measurement system

## Abstract

**Background:**

Inertial hamstring exercises promote functional changes leading to lower rates of hamstring injuries. However, variable training measurement systems have not been specifically validated for hamstring exercises. Accordingly, this study aimed to evaluate the validity of the Inertial Measurement System (IMS) to measure the velocity, force, and power during the performance of different hamstring exercises on a flywheel resistance training device.

**Methods:**

Fifteen males (average age: 22.4 ± 2.5 years; body mass: 77.3 ± 9.8 kg; height: 179.5 ± 7.4 cm; weekly physical activity: 434.0 ± 169.2 min; years of strength training: 4.3 ± 2.2 years) performed the bilateral stiff-leg deadlift (SDL), 45° hip extension (HE), and unilateral straight knee bridge (SKB) in two sessions (familiarization and evaluation) with a 1-week interval between them. The velocity, force, and power (average and peak values) in the concentric and eccentric phases for each of the exercises were recorded simultaneously with IMS and MuscleLab.

**Results:**

Consistency between IMS and MuscleLab was good to excellent for all variables, with *r* ranges from 0.824 to 0.966 in SDL, from 0.822 to 0.971 in HE, and from 0.806 to 0.969 in SKB. Acceptable levels of agreement between devices were observed in general for all exercises, the “bias” ranging from 1.1% to 13.2%. Although MuscleLab showed higher values than IMS for peak velocity, force and power values, the effect size was only relevant for 5 of the 36 parameters. IMS is a new and valid system to monitor inertial hamstring exercises on a new flywheel device. In this way, IMS could have potential practical applications for any professional or athlete who wants to monitor inertial hamstring exercises.

## Introduction

Inertial training is mainly characterized by high intensity and high velocity eccentric contractions (Mosteiro-Muñoz & Domínguez, 2017), generally produced by inertial training devices that use the principle of flywheel (Maroto-Izquierdo et al., 2017). These devices produce unlimited resistance during coupled concentric and eccentric muscle movements using the inertia of a rotating wheel (Maroto-Izquierdo et al., 2017; Weakley et al., 2019). This allows more emphasis to be placed on the eccentric part of an exercise compared to performing it using the traditional weight-stack, a difference that has resulted in greater architectural adaptations for inertial training (Presland et al., 2020). Inertial training has also been shown to be superior to gravitational exercises in generating greater muscle adaptation (Maroto-Izquierdo et al., 2017), with greater improvements in both concentric and eccentric force, muscle power, and muscle hypertrophy (Romero-Rodriguez, Gual & Tesch, 2011; Naczk et al., 2016; Maroto-Izquierdo, García-López & De Paz, 2017).

Inertial training devices usually simulate common gravity-dependent exercise systems such as leg extension (Martinez-Aranda & Fernandez-Gonzalo, 2017), leg curl (Askling, Karlsson & Thorstensson, 2003; Tous-Fajardo et al., 2006; De Hoyo et al., 2015), leg press (Maroto-Izquierdo, García-López & De Paz, 2017), or squat (De Hoyo et al., 2015). However, since the inertial load is not constant, it is difficult to quantify essential variables for training. While the velocity of movement can be measured using various technologies (e.g., linear encoders, optical motion sensing system, or smartphone applications) (Mitter et al., 2019; Pérez-Castilla et al., 2019), the estimation of force and power are conditioned, since inertia from the concentric action directly affects force in the eccentric action (Weakley et al., 2019). Thus, force and power are commonly measured by combining several sensors (e.g., force gauges and linear encoders), calculating power as the product of force and velocity (Tous-Fajardo et al., 2006; Martinez-Aranda & Fernandez-Gonzalo, 2017).

To facilitate the quantification of training load, monitoring devices that track flywheel rotation and transmit live kinetic data have been developed (Bollinger et al., 2018; Weakley et al., 2019), which calculate force and power by relating velocity to a constant moment of inertia. These sensors have been validated only for the squat in a vertical traction system (Bollinger et al., 2018; Weakley et al., 2019), and therefore, the range of exercises in which it can be used is limited. Thus, new alternatives of inertial devices have emerged, such as the EPTE Inertial Concept with its Inertial Measuring System (IMS) sensor, allowing the performance of a greater number of exercises by using different arms and pulleys.

Inertial training has been used both to enhance athletic performance, increased jump height and running speed, producing positive adaptations in risk factors related to hamstring and anterior cruciate ligament injuries (Monajati et al., 2018). In particular, inertial hamstring exercises have been shown to optimize eccentric activation timing and improve absorption and storage of elastic energy for impulse during takeoff, causing functional changes in acceleration and deceleration tasks during sprints with changes of direction (Maroto-Izquierdo, García-López & De Paz, 2017) and reducing the incidence of hamstring injuries (Askling, Karlsson & Thorstensson, 2003; De Hoyo et al., 2015). However, while hamstring training programs recommend exercises based on the activation of the biceps femoris (e.g., bilateral stiff-leg deadlift (SDL), 45° hip extension (HE), or unilateral straight knee bridge (SKB)) (Bourne et al., 2017), since this is the most injured belly of the hamstrings (Verrall et al., 2003), leg curl has been the usual inertial exercise used in order to reduce hamstring injuries (Askling, Karlsson & Thorstensson, 2003; De Hoyo et al., 2015). Even so, prior to the implementation of these new exercises for inertial modality, sensors that can monitor essential variables for training are necessary. Therefore, the aim of this study was to evaluate the validity of the IMS to measure the velocity, force, and power during the performance of different hamstring exercises on a flywheel resistance training device.

## Materials and Methods

### Study design

This study was designed to determine the concurrent validity and agreement of the Encoder EPTE® IMS (Ionclinics SL, L’Alcudia, Spain) to measure velocity, force, and power during different hamstring exercises, using as reference measurement the linear encoder and force gauge of the MuscleLab 4020e (Ergotest Technology AS, Porsgrunn, Norway) (Tous-Fajardo et al., 2006) and its Data Synchronization Unit (DSU) ML6000. This device has been used before as a measurement reference system in inertial training (Tous-Fajardo et al., 2006). All participants completed two sessions with a 1-week interval between sessions. Prior to each session, subjects were instructed not to participate in any strenuous exercise during the preceding 72 h. In the first session, subjects’ anthropometry was measured and they familiarized themselves with the exercises. In the second session, the velocity, force, and power in the concentric and eccentric phases for each of the exercises were recorded simultaneously with IMS and MuscleLab.

### Participants

Fifteen males (average age: 22.4 ± 2.5 years; body mass: 77.3 ± 9.8 kg; height: 179.5 ± 7.4 cm; weekly physical activity: 434.0 ± 169.2 min; years of strength training: 4.3 ± 2.2 years) were evaluated, all of them being volunteers from the University of Valencia. All subjects were currently resistance trained, including recreationally trained individuals and powerlifters, but flywheel naive. They were excluded in the event of injury, disease or pain that could reduce their maximal effort. The experimental protocol was approved by the Ethics Committee of the University of Valencia (Spain) (H1551979435533). Once the study procedures had been explained to the participants in detail, they signed an informed consent and completed the demographic information sheet prior to data collection.

### Procedures

**Figure 1:**
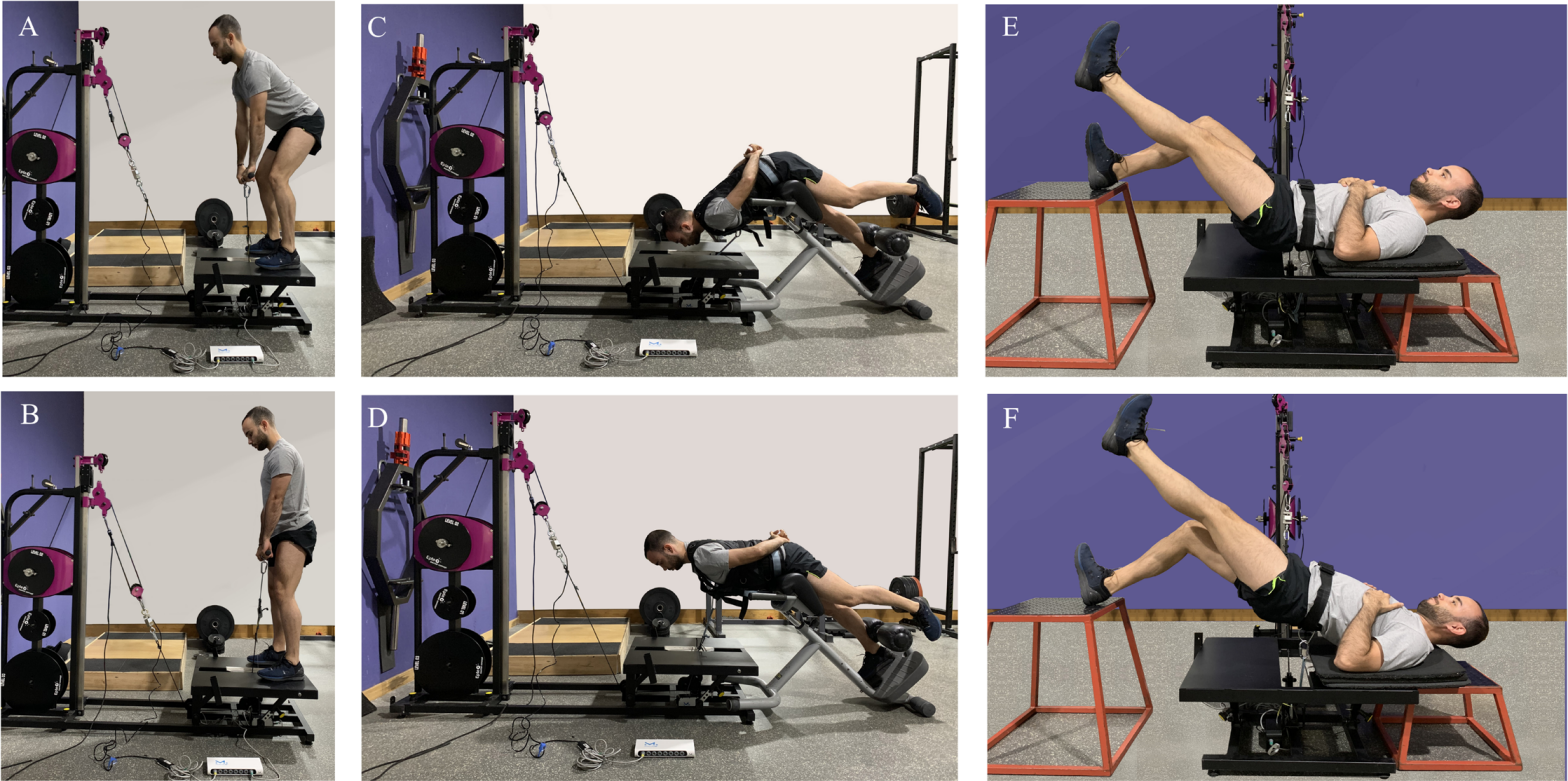
Inertial hamstring exercises: (A and B) bilateral stiff-leg deadlift, (C and D) 45° hip extension, (E and F) unilateral straight knee bridge.

Before each session, subjects completed a 10-min warm-up on a bicycle ergometer at a comfortable speed (80 revolutions per minute) with low resistance (Martín-San Agustín et al., 2020). Exercises performed included SKB, HE, and SDL (Fig. 1) and the testing order was counterbalanced. These exercises were selected to show a high muscular activation of the biceps femoris in relation to the semitendinosus muscle (Bourne et al., 2017). Each exercise started in a concentric phase, with the rope wound on the flywheel axis, and in this position, subjects were instructed to complete 1 warm-up repetition to increase momentum of the flywheel followed by 5 maximal effort repetitions (Bollinger et al., 2018). We instructed participants to apply maximum force during the concentric phase and resist the opposition force during the eccentric phase (Monajati et al., 2018). The initial inertial load for each exercise was selected based on a pilot test conducted beforehand on 10 subjects. The test consisted in obtaining the highest peak power for each exercise, allowing 3–5 attempts. Thus, the inertial load was increased until there was a loss of the peak power of the series, with 5 and 10 min rest between sets. The series with the highest peak power was selected for subsequent analysis.

All the exercises were performed with the EPTE Inertial Concept, an inertial load system which combines a series of exit pulleys and telescopic arms, allowing the performance of multiple lower and upper limb exercises. In addition, interchangeable flywheels (2 × 0.0095 kg·m^−2^, 2 × 0.0472 kg·m^−2^, 2 × 0.151 kg·m^−2^) make it easier to obtain the right inertia for each exercise. The IMS sensor is a quadrature rotary encoder located on the secondary output pulley, with a resolution of 48 points per revolution (4.8 mm) and a time resolution of 0.5 µs. From the time elapsed between pulses, the linear velocity and its variation per unit of time, the acceleration, were calculated. The angular velocity of the inertia axis was calculated from the linear velocity and the effective radius. Rotational kinetic energy was calculated with the angular velocity and the moment of inertia of the disks used. Finally, the power was obtained as the variation of the rotational kinetic energy per unit of time and the force dividing the power by the linear velocity. IMS differentiates the concentric from the eccentric phase according to the direction of rotation of the pulley.

In addition to simultaneously measuring velocity, force, and power with the IMS and the MuscleLab sensors (i.e., linear encoder and force gauge), the force gauge was anchored along the line of the rope, between a pulley and the proximal end of the second rope. In turn, the linear encoder was placed parallel to the stretch of rope running from the last pulley to the grip or harness (Fig. 1). The gauge and encoder were connected to the DSU to synchronize their information. Thus, the velocity and the acceleration were obtained using the linear encoder and the force value with the gauge, and the power was calculated combining both sensors. The average and peak values for velocity, force, and power by phase were calculated using custom written scripts computed with MATLAB (version R2019b; The Mathworks, Natick, MA, USA). Concentric and eccentric phases were identified with the linear encoder (i.e., positive velocity for the concentric phase and negative for the eccentric).

### Statistical analysis

Participant characteristics and velocity, force, and power values (m/s, N, or W, respectively) by concentric and eccentric phase are presented as average (SD) or percentages, as appropriate. Mean between repetitions was used for analyses. For all analyses, we used SPSS (version 24; SPSS Inc., Chicago, IL, USA) and MedCalc Statistical Software (MedCalc Software, Mariakerke, Belgium) to build the Bland–Altman graphs.

Validity is defined as the extent to which the method measures what it is intended to measure (Streiner & Norman, 2008). In this study concurrent validity was used, which is a type of criterion validity and which it is used when the tool being validated conducts simultaneous measurements (Pourahmadi et al., 2019). Agreement between measurements refers to the degree of concordance between two (or more) sets of measurements (Ranganathan, Pramesh & Aggarwal, 2017).

To analyze the concurrent validity and the agreement between instruments for velocity, force, and power values (peaks and averages) by concentric and eccentric phases for SDL, HE, and SKB, Pearson’s product-moment correlation coefficient (*r*) and Bland–Altman plots were used, respectively. Criteria for validity were defined a priori as good (0.700–0.799), very good (0.800–0.899), or excellent (*r* > 0.900), whereas *r* < 0.700 was considered unacceptable (Bollinger et al., 2018). Additionally, the following values were calculated: the upper and lower limits of agreement (LoA), the mean, and SD of the difference between sensors (both absolute difference and percentages) regarding the MuscleLab values. The latter two statistics are respectively called bias and imprecision. The standard error (SE) of the difference was also calculated. Bland–Altman plots were generated to illustrate the agreement between devices for peak velocity in both phases. Paired *t*-tests were also used to analyze differences between devices and Cohen’s *d* was calculated to evaluate the effect size (*d*, 0.2: trivial, 0.2–0.5: small, 0.5–0.8: medium, and >0.8: large) (Cohen, 1977).

## Results

Both devices showed good to excellent correlations for all variables, with *r* ranges from 0.824 to 0.966 for SDL (Table 1), from 0.822 to 0.971 for HE (Table 2), and from 0.806 to 0.969 for SKB (Table 3).

**Table 1:** Validity between IMS and MuscleLab to measure the peak and average velocity, force, and power by concentric and eccentric phase in the stiff-leg deadlift.

	Concentric							Eccentric						
	MuscleLab (SD)	IMS (SD)	Pearson’s *r*	LoA−/LoA+	Mean difference (%); effect size	SD difference (%)	SE difference	MuscleLab (SD)	IMS (SD)	Pearson’s *r*	LoA−/LoA+	Mean difference (%); effect size	SD difference (%)	SE difference
Velocity (m/s)														
Peak	0.95 (0.11)	0.91 (0.19)	0.964	−0.14/0.06	−0.04 (−4.2%); 0.23*	0.05 (5.3%)	0.01	1.04 (0.23)	0.97 (0.21)	0.965	−0.18/0.05	−0.07 (−6.7%); 0.31*	0.06 (5.8%)	0.01
Average	0.57 (0.14)	0.62 (0.13)	0.910	−0.06/0.17	0.05 (8.8%); 0.39*	0.06 (10.5%)	0.01	0.59 (0.12)	0.63 (0.15)	0.960	−0.05/0.13	0.04 (6.8%); 0.30*	0.05 (8.5%)	0.01
Force (N)														
Peak	1140.7 (164.3)	1029.0 (159.5)	0.917	−241.2/17.9	−111.6 (−9.8%); 0.69*	66.1 (5.8%)	17.1	1046.7 (218.4)	918.4 (197.4)	0.851	−354.1/97.4	−128.4 (−12.3%); 0.62*	115.2 (11%)	29.7
Average	881.9 (137.2)	855.9 (86.7)	0.824	−186.8/134.9	−25.9 (−2.9%); 0.23	82.1 (9.3%)	21.2	636.2 (121.4)	643.5 (79.1)	0.845	−127.9/142.6	7.3 (1.1%); 0.07	69.0 (10.8%)	17.8
Power (W)														
Peak	1003.7 (273.3)	939.7 (217.4)	0.941	−261.2/133.2	−63.9 (−6.4%); 0.26*	100.6 (10%)	25.9	721.4 (257.5)	688.7 (232.8)	0.941	−204.5/139.1	−32.7 (−4.5%); 0.13	87.6 (12.1%)	22.6
Average	525.1 (183.6)	463.8 (155.6)	0.966	−163.9/41.2	−61.3 (−11.7%); 0.36*	52.3 (10%)	13.5	385.5 (129.4)	365.9 (125.4)	0.910	−125.6/86.43	−19.57 (−5.1%); 0.15	54.1 (14%)	13.9

**Notes:**

IMS: Inertial Measure System; SD: Standard deviation; LoA: Limit of agreement; SE: Standard error.

* Significant differences between devices.

**Table 2:** Validity between IMS and MuscleLab to measure the peak and average velocity, force, and power by concentric and eccentric phase in the 45° hip extension.

	Concentric							Eccentric						
	MuscleLab (SD)	IMS (SD)	Pearson’s *r*	LoA−/LoA+	Mean difference (%); effect size	SD difference (%)	SE difference	MuscleLab (SD)	IMS (SD)	Pearson’s *r*	LoA−/LoA+	Mean difference (%); effect size	SD difference (%)	SE difference
Velocity (m/s)														
Peak	0.55 (0.12)	0.53 (0.11)	0.971	−0.06/0.04	−0.02 (−3.6%); 0.11	0.03 (5.5%)	0.01	0.64 (0.14)	0.56 (0.12)	0.915	0.19/0.02	−0.08 (−12.5%); 0.66*	0.05 (7.8%)	0.01
Average	0.33 (0.07)	0.37 (0.08)	0.866	−0.04/0.11	0.04 (12.1%); 0.54*	0.04 (12.1%)	0.01	0.36 (0.08)	0.39 (0.08)	0.895	0.04/0.1	0.03 (8.3%); 0.38*	0.04 (11.1%)	0.01
Force (N)														
Peak	597.8 (121.3)	560.4 (159.1)	0.871	−194.3/119.4	−37.4 (−6.3%); 0.26	80 (13.4%)	20.7	546.2 (148.7)	488.8 (148.8)	0.923	171.7/56.9	−57.4 (−10.5%); 0.38*	58.3 (10.7%)	15.1
Average	337.3 (74.9)	271.7 (88.2)	0.863	−153/21.8	−44.6 (−13.2%); 0.79*	44.6 (13.2%)	11.5	217.7 (58.1)	198.9 (50.2)	0.867	77.1/26.2	−25.4 (−11.7%); 0.49*	26.3 (11.7%)	6.8
Power (W)														
Peak	193.3 (55.7)	171.5 (56.4)	0.822	−87.4/43.8	−21.8 (−11.3%); 0.39*	33.5 (17.3%)	8.6	133.9 (36.9)	143.2 (47.6)	0.835	42.4/60.9	9.3 (6.9%); 0.22	26.3 (19.6%)	6.8
Average	100.4 (38.7)	89.5 (34.4)	0.894	−44.7/23.1	−10.8 (−10.8%); 0.30*	17.3 (17.2%)	4.5	68.6 (19.1)	76.9 (23.4)	0.904	11.6/28.4	8.4 (12.2%); 0.39*	10.2 (14.9%)	2.6

**Notes:**

IMS: Inertial Measure System; SD: Standard deviation; LoA: Limit of agreement; SE: Standard error.

* Significant differences between devices.

**Table 3:** Validity between IMS and MuscleLab to measure the peak and average velocity, force, and power by concentric and eccentric phase in the unilateral straight knee bridge.

	Concentric							Eccentric						
	MuscleLab (SD)	IMS (SD)	Pearson’s *r*	LoA−/LoA+	Mean difference (%); effect size	SD difference (%)	SE difference	MuscleLab (SD)	IMS (SD)	Pearson’s *r*	LoA−/LoA+	Mean difference (%); effect size	SD difference (%)	SE difference
Velocity (m/s)														
Peak	0.46 (0.15)	0.47 (0.13)	0.957	−0.08/0.10	0.01 (2.2%); 0.07	0.04 (8.7%)	0.01	0.64 (0.16)	0.57 (0.14)	0.947	−0.16/0.03	−0.06 (−9.4%); 0.43*	0.05 (7.8%)	0.01
Average	0.31 (0.08)	0.35 (0.10)	0.922	−0.04/0.12	0.04 (12.9%); 0.41*	0.04 (12.9%)	0.01	0.33 (0.09)	0.34 (0.09)	0.854	−0.08/0.11	0.01 (3.0%); 0.17	0.05 (15.2%)	0.01
Force (N)														
Peak	670.2 (154.4)	599.3 (140.9)	0.953	−163.4/21.6	−70.9 (−10.6%); 0.48*	47.2 (7%)	12.2	491.9 (151.9)	428.9 (134.1)	0.958	−151.6/25.7	−63.0 (−12.8%); 0.44*	45.2 (9.2%)	11.7
Average	406.1 (100.2)	371.7 (85.1)	0.951	−98.3/29.6	−34.4 (−8.5%); 0.37*	32.6 (8%)	8.4	199.4 (95.4)	195.9 (79.1)	0.934	−73.0/66	−3.5 (−1.8%); 0.04	35.5 (17.8%)	9.2
Power (W)														
Peak	220.1 (180.7)	181.7 (128.8)	0.969	−164.9/87.9	−38.4 (−17.4%); 0.24*	64.5 (29.3%)	16.6	149.4 (106.4)	158.9 (79.2)	0.806	−133.6/114.7	9.5 (6.4%); 0.10	63.4 (42.4%)	16.4
Average	88.4 (86.6)	90.3 (63.9)	0.917	−72.2/76.1	1.9 (2.1%); 0.02	37.8 (42.8%)	9.8	73.8 (21.1)	72.4 (19.7)	0.898	−19.7/16.7	−1.5 (−2.0%); 0.07	9.3 (12.6%)	2.4

**Notes:**

IMS: Inertial Measure System; SD: Standard deviation; LoA: Limit of agreement; SE: Standard error.

* Significant differences between devices.

Acceptable levels of agreement between devices were observed in general for all exercises, the “bias” ranging from 1.1% to 13.2%, except for the concentric power peak in SKB (17.4%). Overall, MuscleLab showed higher values than IMS for peak velocity, force and power values, but the effect size was only relevant for 5 of the 36 parameters: force peaks for SDL and average velocity and average force in the concentric phase and eccentric velocity peak for HE. Other parameters also exhibited significant differences but effect sizes from trivial to small.

**Figure 2:**
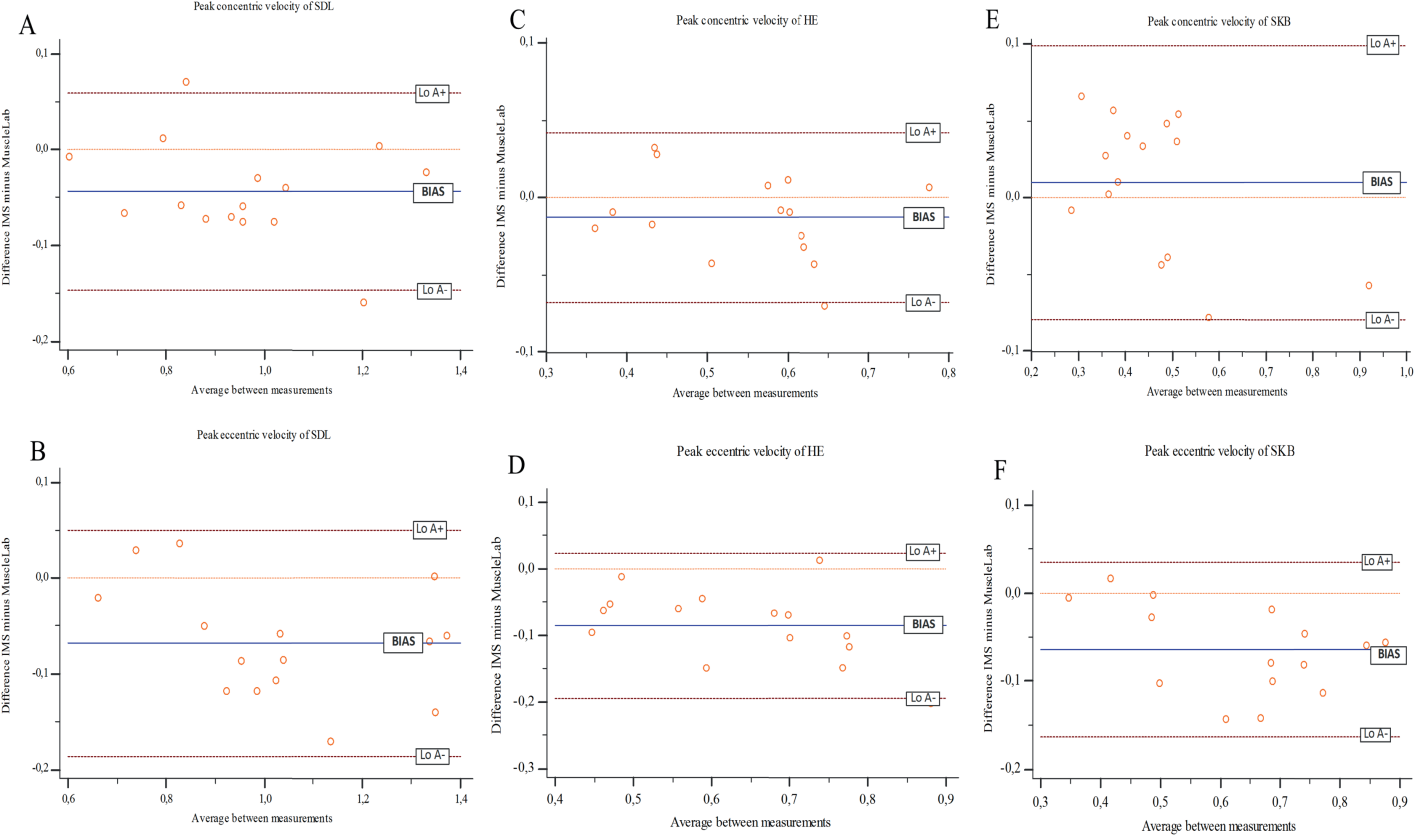
Bland–Altman plots for IMS and MuscleLab for: (A) concentric and (B) eccentric velocity peak in the bilateral stiff-leg deadlift (SDL), (C) concentric and (D) eccentric velocity peak in the 45° hip extension (HE), (E) concentric and (F) eccentric velocity.

Furthermore, ‘imprecision’ values were acceptable, ranging between 5.5% and 15.2% for most of the parameters, except for the power values of HE and SKB (14.9–19.6% and 12.6–42.8%, respectively). Still, most of the subjects were within the “imprecision” values, with few subjects (around 3–4) between one SD and the LoAs obtained (Fig. 2).

## Discussion

The present study analyzed IMS validity for measuring the velocity, force, and power of hamstring exercises performed on a flywheel resistance training device. While all the variables in both concentric and eccentric phases showed good to excellent Pearson’s *r* values, acceptable levels of agreement, and trivial-small effect size for most parameters, power values of HE and SKB showed large imprecision. Therefore, IMS is a valid sensor to measure most of the variables analyzed, although power values in HE and SKB should be taken with caution.

As in traditional training methods, load quantification in inertial training is needed to assess the physical stress and direct the stimuli appropriately (Weakley et al., 2019). While in traditional methods the load is mainly evaluated in the concentric phase, inertial training also requires variable monitoring in the eccentric phase. Since the eccentric component of inertial training appears to be responsible for greater skeletal muscle adaptations (i.e., force, power and muscle mass) as compared to gravity-dependent training, valid evaluation methods in both phases are needed.

To our knowledge, this is the first study to examine the concurrent validity of an inertial sensor in multiple exercises per concentric and eccentric phases. Our findings are consistent with those from previous studies that examined the validity of an inertial sensor for squat (kMeter) (Weakley et al., 2019), which also showed good to excellent correlations with acceptable levels of agreement. While squat and SDL are vertical-force vector exercises, HE and SKB are performed along a diagonal vector. Accordingly, IMS is able to measure inertial training variables regardless of the direction of the vector.

Inertial training of the hamstrings has been proposed as a new alternative for injury prevention (Tous-Fajardo et al., 2006), since its eccentric emphasis directly influences intrinsic risk factors to hamstring injury such as poor eccentric force of the hamstrings (Schache et al., 2012; Yu, Liu & Garrett, 2017) or short length of a biceps femoris fascicle (Presland et al., 2020). While leg curl, squat, hip extension, or Romanian deadlift have been studied as inertial hamstring exercises (Tous-Fajardo et al., 2006; Bollinger et al., 2018; Monajati et al., 2018), other gravity-dependent exercises that have been shown to further activate the biceps femoris (Bourne et al., 2017) have not been used in inertial training. Therefore, our findings provide valuable information on a flywheel resistance training device for the IMS-monitored performance of selective biceps activation exercises (SDL, HE and SKB).

To the best of our knowledge, no previous studies have examined the validity of a sensor to quantify inertial variables for multiple exercises per concentric and eccentric phase. Furthermore, the use of a gold standard measure and trained resistance participants could be considered strengths of the study. Despite its novel findings, this study was subject to some limitations. First, IMS validity was only tested for the measurement of inertial training variables, but the reliability of the protocol was not evaluated. Previous studies have shown the reliability of inertial measures for each individual lift and for each outcome of interest (Bollinger et al., 2018). Since our protocol aims to obtain maximum power per exercise with a large amount of inertia levels (26 levels resulting from the combination of all the discs), this could possibly limit the study of stability over time. In addition, participants had no flywheel experience, and therefore learning effects may have influenced the reliability of measurements. Thus, future studies should use participants who regularly use inertial training and previously select the study inertias. Secondly, although all variables showed good to excellent values for *r* and “bias” showed an acceptable level of agreement, as with kMeter (Weakley et al., 2019), the differences between devices suggest that caution should be taken when using inertial measures for research purposes.

## Conclusions

Our study proposes a new valid system to monitor inertial hamstring exercises in a flywheel resistance training device, including for the first time specific exercises for the biceps femoris. Our findings prove that the IMS is valid to measure speed and force in the exercises studied and power in the SDL, but contrary to that, various power parameters in HE and SKB does not achieve the minimum validity. Therefore, IMS could have potential practical applications for any professional or athlete who wants to monitor inertial hamstring exercises.

## Supplemental Information

10.7717/peerj.10169/supp-1 Supplemental Information 1 Database.

10.7717/peerj.10169/supp-2 Supplemental Information 2 MatLab Script.

## Additional Information and Declarations

### Data Availability

The following information was supplied regarding data availability: The raw data and Matlab code are available as Supplemental Files.
